# Diplopia: A Rare Manifestation of Neuroborreliosis

**DOI:** 10.1155/2018/9720843

**Published:** 2018-07-09

**Authors:** Ayushi Dixit, Yesika Garcia, Lauren Tesoriero, Charles Berman, Vincent Rizzo

**Affiliations:** ^1^NYC Health + Hospitals/Queens, Icahn School of Medicine at Mount Sinai, USA; ^2^New York Institute of Technology College of Osteopathic Medicine, USA

## Abstract

Early disseminated Lyme disease typically presents with cardiac, rheumatologic, or neurologic symptoms. Though uncommon,* Borrelia burgdorferi* can invade the central nervous system and cause neuroborreliosis. In these patients, facial palsy, headache, and stiffness of the neck are the most common presenting symptoms. Our case describes a patient with oculomotor nerve palsy manifesting as double vision as the initial presentation of neuroborreliosis.

## 1. Introduction

Lyme disease is caused by the spirochete* Borrelia burgdorferi* and is the most common vector-borne disease in the United States. It most commonly presents with erythema migrans or flu-like symptoms. Neuroborreliosis is a term used to describe Lyme disease of the central nervous system. The most common complications of neuroborreliosis include meningitis, facial nerve palsy, and peripheral neuropathy. Diagnosis of the disease is usually clear when the patient recalls a tick bite and/or rash before the onset of symptoms. We present a case of neuroborreliosis manifesting with diplopia.

## 2. Case Presentation

A 69-year-old male with past medical history of type 2 diabetes and hypertension presented to the emergency department in New York City in August complaining of headache and diplopia. His headache abruptly began one week ago, was localized to the right occipital region, and gradually moved to his right orbit. Five days later he developed diplopia. One month prior to symptom onset, he hiked in a rural area of New York State, but he denied any tick bites or rash development. On presentation, our patient was hemodynamically stable, did not have any signs of acute infection, and denied any fevers or chills. He stated he had double vision when opening both eyes; however if he covered his right eye his vision normalized. Physical exam was significant for left sided cranial nerve 3 palsy. The rest of his physical and neurological examinations were normal. MRI and MRA were both negative. Syphilis serology was negative. Lumbar showed glucose of 101, protein of 77, and 74 white blood cells (84% lymphocytes and atypical lymphocytes). CSF was negative for VDRL, cryptococcal antigen, varicella zoster, HSV 1 and 2, and West Nile virus. He had positive Lyme titers by ELISA at 6.04 (negative < 0.90) and western blot showed five IgG and two IgM bands. He was started on acyclovir and ceftriaxone and experienced resolution of headache but continued to complain of diplopia. Lyme antibody in CSF was checked by ELISA and was reactive at 0.532 (reactive cutoff 0.144). Although the CSF-to-serum ratio of IgG by Eliza was only 0.0880, patient was treated for oculomotor nerve palsy secondary to Lyme meningitis. Acyclovir was discontinued. He was treated with ceftriaxone for four weeks as per The Sanford Guide to Antimicrobial Therapy guidelines. His diplopia resolved and he was asymptomatic two months after initiation of therapy.

## 3. Conclusion

This case is an example of a rare presentation of neuroborreliosis. Although Lyme is known to affect the neurological system it usually does not manifest in the extraocular muscles. Approximately three-fourths of patients with Lyme-associated cranial neuropathies present with a facial nerve palsy [1]. Lyme disease related ocular complications are uncommon, but various manifestations have been described including conjunctivitis, keratitis, and extraocular muscle palsies [2]. Additionally, few cases of optic nerve papillitis have been reported [3]. Upon our review very few cases of Lyme disease affecting extraocular muscles have been reported in literature and we were unable to find any that highlighted that the third cranial nerve alone, as seen in our patient, was affected.

## 4. Discussion

Nervous system involvement occurs in up to fifteen percent of patients with untreated* B. burgdorferi *infection [4]. Patients with early Lyme neuroborreliosis usually present in the summer and early fall, with cranial neuropathy, particularly seventh nerve palsy [5]. In our case the third cranial nerve was the only nerve affected. Although neuroborreliosis is commonly associated with facial nerve palsy it may account for otherwise unexplained neurological manifestations and warrants evaluation with lumbar puncture and CSF studies. Treatment with recommended antibiotics is effective in Lyme neuroborreliosis, and patients with early disease usually have excellent outcomes. Recovery is slower and may be incomplete in patients with late disease [1]. Patients with unexplained symptoms and lab findings prompt further investigation of history in detail. It has been speculated that only about twenty-five percent of patients with Lyme disease recall a tick bite [6], further stressing the importance of an in-depth history and physical exam, with suspicion in patients visiting endemic regions. Identifying the early Lyme disease is vital for immediate treatment to prevent worsening and chronicity of disease.
